# Cost-effectiveness of Artificial Intelligence for Proximal Caries Detection

**DOI:** 10.1177/0022034520972335

**Published:** 2020-11-16

**Authors:** F. Schwendicke, J.G. Rossi, G. Göstemeyer, K. Elhennawy, A.G. Cantu, R. Gaudin, A. Chaurasia, S. Gehrung, J. Krois

**Affiliations:** 1 Department of Oral Diagnostics, Digital Health and Health Services Research, Charité–Universitätsmedizin Berlin, Berlin, Germany; 2 Department of Operative and Preventive Dentistry, Charité–Universitätsmedizin Berlin, Berlin, Germany; 3 Department of Orthodontics, Dentofacial Orthopedics and Pedodontics, Charité–Universitätsmedizin Berlin, Berlin, Germany; 4 Department of Oral and Maxillofacial Surgery, Charité-Universitätsmedizin Berlin, Berlin, Germany; 5 Department of Oral Medicine and Radiology, King George’s Medical University, Lucknow, India

**Keywords:** caries diagnosis/prevention, computer simulation, dental, decision making, economic evaluation, radiology

## Abstract

Artificial intelligence (AI) can assist dentists in image assessment, for example, caries detection. The wider health and cost impact of employing AI for dental diagnostics has not yet been evaluated. We compared the cost-effectiveness of proximal caries detection on bitewing radiographs with versus without AI. U-Net, a fully convolutional neural network, had been trained, validated, and tested on 3,293, 252, and 141 bitewing radiographs, respectively, on which 4 experienced dentists had marked carious lesions (reference test). Lesions were stratified for initial lesions (E1/E2/D1, presumed noncavitated, receiving caries infiltration if detected) and advanced lesions (D2/D3, presumed cavitated, receiving restorative care if detected). A Markov model was used to simulate the consequences of true- and false-positive and true- and false-negative detections, as well as the subsequent decisions over the lifetime of patients. A German mixed-payers perspective was adopted. Our health outcome was tooth retention years. Costs were measured in 2020 euro. Monte-Carlo microsimulations and univariate and probabilistic sensitivity analyses were conducted. The incremental cost-effectiveness ratio (ICER) and the cost-effectiveness acceptability at different willingness-to-pay thresholds were quantified. AI showed an accuracy of 0.80; dentists’ mean accuracy was significantly lower at 0.71 (minimum–maximum: 0.61–0.78, *P* < 0.05). AI was significantly more sensitive than dentists (0.75 vs. 0.36 [0.19–0.65]; *P* = 0.006), while its specificity was not significantly lower (0.83 vs. 0.91 [0.69–0.98]; *P* > 0.05). In the base-case scenario, AI was more effective (tooth retention for a mean 64 [2.5%–97.5%: 61–65] y) and less costly (298 [244–367] euro) than assessment without AI (62 [59–64] y; 322 [257–394] euro). The ICER was −13.9 euro/y (i.e., AI saved money at higher effectiveness). In the majority (>77%) of all cases, AI was less costly and more effective. Applying AI for caries detection is likely to be cost-effective, mainly as fewer lesions remain undetected. Notably, this cost-effectiveness requires dentists to manage detected early lesions nonrestoratively.

## Introduction

Dental caries affects more than 3 billion individuals globally and generates significant costs and health care burden (Kassebaum et al. 2017). Building on the understanding that initial (noncavitated) caries lesions can be arrested non- or microinvasively (e.g., fluoride varnish application, sealing, caries infiltration), the traditional invasive/restorative therapy of caries is restricted to advanced lesions, mainly as the placement of restorations has been demonstrated to initiate a spiral of escalating and increasingly expensive retreatments (Frencken et al. 2016; Schwendicke, Splieth, et al. 2019).

Early management of noncavitated lesions requires their detection first. The standard diagnostic strategy, visual-tactile inspection, does usually not permit detecting early lesions on nonassessable (e.g., proximal) surfaces (Gimenez et al. 2015). A common additional method to detect early lesions on proximal surfaces and to assess their extent is bitewing radiography (Schwendicke, Stolpe, et al. 2015). While being more sensitive for detecting early lesions than visual-tactile assessment, the assessment of bitewings comes with significant variance between examiners and a considerable proportion of false-positive or false-negative detections (Schwendicke, Stolpe, et al. 2015).

Artificial intelligence (AI), specifically deep learning using convolutional neural networks (CNNs), has been suggested to help overcome the limited reliability and validity of dental image analysis. CNNs allow to map an input (image) to an output (classification), based on a set of weights, learned from data (LeCun et al. 2015). The learning process involves labeling an image and providing both the image and the label to the CNN, which iteratively adjusts itself to eventually be able to predict the presence of the labeled entity (a carious lesion) on unseen data. For detecting caries lesions, we previously trained, validated, and tested a fully convolutional neural network, U-net, yielding an accuracy superior to individual dentists (Garcia Cantu et al. 2020). Notably, the CNN showed significantly higher sensitivity than dentists, especially for detecting early lesions (more details are provided below). Dentists would use this AI technology via a software application, in our case allowing to display detected carious lesions on bitewings provided to the software via an upload function. The clinical flow would be expanded by the radiographic viewing software, for instance, allowing to assess bitewings in their native state and using an overlay of AI-detected lesions (assisting the assessment of lesion extension/depth).

In clinical care, the detection of a pathology (a caries lesion) has only limited impact on the patient (e.g., his or her health) or the health care system (e.g., the generated costs). Health outcomes and costs are instead determined by the subsequent treatment decisions (the costs for the diagnostics themselves are usually only a fraction of the treatment costs). We previously demonstrated that a systematic evaluation of diagnostic strategies should be performed in combination with the provided therapy, ideally over a long-term horizon, mainly as initial treatment decisions come with long-term consequences (e.g., retreatments required and costs generated) (Schwendicke, Paris, et al. 2015; Schwendicke, Stolpe, et al. 2015). In the present study, we aimed to assess the cost-effectiveness of employing AI for proximal caries detection on bitewing radiographs.

## Methods

### Study Design

A model-based cost-effectiveness study was performed, building on a previously conducted diagnostic accuracy study (Garcia Cantu et al. 2020), in which a CNN was trained, validated, and tested on 3,686 retrospectively collected bitewing radiographs from a German dental clinic. Bitewings had been assessed for proximal caries lesions by a total of 4 experts (reference test). Only proximal caries lesions on permanent teeth were included. The yielded accuracy data were used to inform an established cost-effectiveness model (Schwendicke, Paris, et al. 2015). Note that, in a real-life setting, dentists would not only rely on AI but also triangulate the findings with those from clinical assessments and so on. They would further integrate patient-level aspects (e.g., caries risk) and employ a range of further treatments (e.g., fluoride varnish instead of caries infiltration). Reporting of this study follows the Consolidated Health Economic Evaluation Reporting Standards (CHEERS) (Husereau et al. 2013).

### Setting, Perspective, Population, Horizon

This study adopted a mixed public-private-payer perspective in the context of German health care (for more details, see the Appendix). Note that given this perspective, the true costs to clinicians and society (e.g., opportunity costs, nonmedical costs) are not fully reflected. For reimbursement decisions, though, this perspective is most relevant, which is why it has been the most common one in dental health economics in the past.

We modeled a population of posterior permanent teeth in initially 12-y-old individuals using TreeAge Pro 2019 R1.1 (TreeAge Software), with the teeth’s proximal surfaces at the beginning of the simulation being sound, initially carious, or advanced carious according to prevalence data drawn from a previous study (Schwendicke, Paris, et al. 2015). We assumed all teeth to have a vital sensible pulp and not more than 1 lesion to occur per tooth.

### Comparators

We compared 2 detection strategies for proximal caries lesions. In both groups, biannual visual-tactile caries detection was assumed to be performed, allowing to detect advanced (D2, D3) with some accuracy but not initial lesion stages. For these advanced stages, the only treatment available was restorative care (Schwendicke, Paris, et al. 2015).

In the control group (no AI), radiographic caries detection on bitewings by individual dentists was assumed to be provided in addition to visual-tactile detection every 2 y, allowing to detect also initial lesions and to increase the sensitivity to detect advanced ones. The accuracy data informing this group were built on a systematic review and meta-analysis (Schwendicke, Tzschoppe, et al. 2015), assuming this to be the most robust data available. In addition, and for the purpose of sensitivity analyses, we used the diagnostic accuracies of 7 independent dentists who had evaluated the same test data of 141 bitewing radiographs on which the CNN had been tested (Garcia Cantu et al. 2020). In the test group (AI), radiographic caries detection on bitewings provided every 2 y was assumed to be assisted by a diagnostic assistance system, based on a fully convolutional neural net, U-Net (Ronneberger et al. 2015), that had been trained, validated, and tested as described elsewhere (Garcia Cantu et al. 2020). The emanating accuracy differences between groups translated into different treatment decisions and thereby cost differences. For the test groups, costs were further modified by the AI intervention generating costs on a per-use basis (see below).

### Cost-effectiveness Model and Assumptions

**Figure 1.**
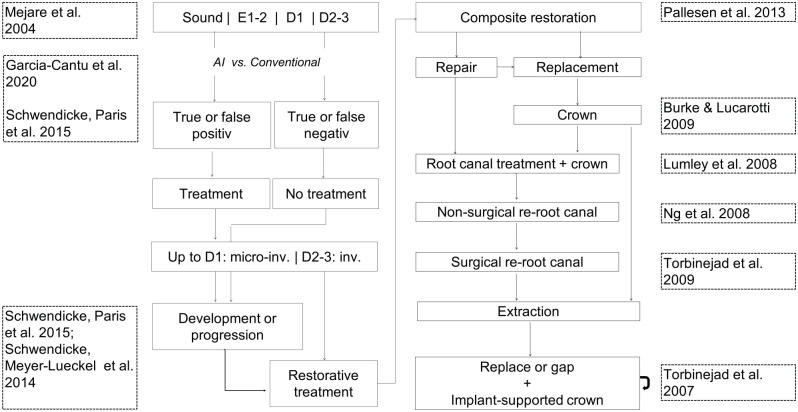
Input data and model. The state diagram (central parts) shows the different health states (solid boxes). Transition or allocation probabilities determined the chance of passing between them, indicated by arrows. The data sources used to simulate individuals’ flow through the model are shown in dotted boxes at the left and right. Individuals started with teeth being either sound or showing E1/2/D1 and D2–D3 lesions. Sound surfaces could be detected as such, without any subsequent treatment, or false positively detected as initial (E2/D1) caries lesions depending on the detection method. False-positive detections on sound surfaces led to infiltration treatment, without any effectiveness gain, but money spent unnecessarily. Initial lesions (E2/D1) could again be detected (treated by resin infiltration) or not detected (and assumed to progress with some chance) and, depending on the efficacy of resin infiltration, be arrested or progress to D2 lesions. In a sensitivity analysis, we assumed all detected lesions to be treated restoratively instead. For advanced lesions (D2) not extending into the inner third of the dentin (D3), a two-surfaced restoration was assumed to be placed. This placement was not to be associated with pulpal risks, mainly as we assumed these lesions to be not deep (no proximity to the pulp). For lesions extending into the inner one-third of the dentin, the risk of pulp exposure was estimated at 0.3, and exposed pulps received direct pulp capping. The risk of restorative complications was derived from previous studies, and if restorations failed, they were assumed to be either renewed or repaired. If failing again, the placement of a full-metal crown (the standard crown therapy for most posterior teeth within statutory German health insurance) was assumed. Failed crowns were assumed to be replaced once, after which the tooth was extracted. Extracted teeth were assumed to be replaced using implant-supported single crowns; the proportion of replaced teeth was 0.8 in the base case and varied in sensitivity analyses.

We used a Markov simulation model, consisting of initial and follow-up health states. We modeled posterior teeth over their lifetime, with sound surfaces, initial carious lesions (E1/2/D1), or advanced ones (D2–D3) being the initial health states, the distribution of which was derived as described below. E1/2/D1 may be detected and therapeutically arrested or not; in this case, they could progress to D2–D3 lesions, which would at some point be restored using a composite restoration (with a risk of endodontic complications for D3 lesions). Restorations could fail and be replaced or repaired, and after repeated failure, a crown was to be placed, which again could fail and be replaced once. Similarly, endodontic complications could occur, which would be treated using root canal treatment, followed by nonsurgical and eventually surgical retreatment in case of endodontic complications. If no further restorative or endodontic treatment option remained, an extraction was assumed, and teeth were replaced (with a certain probability) using implant-supported single crowns (ISCs, with both the implant and the crown coming with risks). The possibility of teeth transitioning to the next health state was based on transition probabilities. Simulation was performed in discrete annual cycles. The model (Fig. 1) had been previously employed and validated (Schwendicke, Paris, et al. 2015).

### Input Variables

The sources of accuracy data have been described; data on prevalence are described in the Appendix. Further transition probabilities were largely built on data used in previous studies and the therein included calculations, as described in Table 1. Notably, the used data stemmed mainly from large cohort studies or systematic reviews (i.e., showed robustness), and their validity has been demonstrated before (Schwendicke et al. 2013; Schwendicke, Graetz, et al. 2014; Schwendicke, Meyer-Lueckel, et al. 2014; Schwendicke, Paris, et al. 2015; Schwendicke, Stolpe, et al. 2015).

**Table 1.** Input Parameters.

Prevalence, Accuracy, Lesion Development, and Progression				
Estimate	Source (Reference)	Lesions into Inner Third of Enamel (E2)	Lesions into Outer Third of Dentin (D1)	Lesions into Middle Third of Dentin (D2)
Prevalence				
Low risk	Schwendicke, Paris, et al. 2015	0.14	0.025	0.005
High risk	Schwendicke, Paris, et al. 2015	2.14 × 0.14	1.66 × 0.025	1.66 × 0.005
Sensitivity and specificity				
Sensitivity visual-tactile	Schwendicke, Paris, et al. 2015	0.00	0.00	0.311 (0.270–0.353)
Specificity visual-tactile	Schwendicke, Paris, et al. 2015	1.00	1.00	0.922 (0.892–0.945)
Sensitivity radiography without AI (control)^a^	Schwendicke, Tzschoppe, et al. 2015	0.24 (0.21–0.26)	0.36 (0.24–0.49)	0.64 (0.59–0.70)
Specificity radiography without AI (control)^a^	Schwendicke, Tzschoppe, et al. 2015	0.97 (0.95–0.98)	0.94 (0.89–0.97)	0.98 (0.97–0.98)
Sensitivity radiography with AI (test)	Garcia Cantu et al. 2020	0.68	0.68	0.58
Specificity radiography with AI (test)	Garcia Cantu et al. 2020	0.86	0.86	0.96
Probability of lesion development	Schwendicke, Paris, et al. 2015	*P* = 1.26 × 0.57252 × 2.7^−0.1472 × 2α^ distribution: 1.24–1.29	*P* = 1.26 × 0.0426 × 2.7^−0.0521 × 2α^ distribution: 1.24–1.29	*P* = 1.26 × 0.57 × 0.0426 × 2.7^−0.0521 × 2α^ distribution: 1.24–1.29
Probability of lesion progression				
Progression to		D1 lesion	D2 lesion	D3 lesion
If untreated	Schwendicke, Paris, et al. 2015	*P* = 2.63 (high risk) / 2.13 (low risk) × 3.0984 × (2α)^−1.343^ (distribution: *P* × 0.87 – *P* × 1.13)	*P* = 2.63 (high risk) / 2.13 (low risk) × 161.52 × (2α)^−2.078^ (distribution: *P* × 0.87 – *P* × 1.13)	*P* = 1.32 × 161.52 × (2α)^−2.078^ (distribution: *P* × 0.87 – *P* × 1.13)
If infiltrated	Schwendicke, Paris, et al. 2015	*P* = 0.4289 × (2α)^−1.391^ (distribution: *P* × 0.23 – *P* × 5.15)	*P* = 68.869 × (2α)^−2.078^ (distribution: *P* × 0.23 – *P* × 4.17)	N/A
Transition Probabilities				
Health State	Source (Reference)	Transition Probability per Cycle	Transition to	Allocation Probability
Composite^b^	Pallesen et al. 2013	0.016	Composite	0.45
			Crown	0.10
			Repair	0.10
			Root canal treatment	0.25
			Extraction	0.10
Direct capping^c^	Schwendicke et al. 2013	0.111	Root canal treatment	0.95
			Extraction	0.05
Crown on vital tooth^d^	Burke and Lucarotti 2009	0.036	Root canal treatment	0.25
			Recementation	0.15
			Repair	0.10
			Recrown	0.40
			Extraction	0.10
Root canal treatment	Lumley et al. 2008	0.021	Nonsurgical retreatment	0.20
			Surgical retreatment	0.30
			Extraction	0.50
Crown on nonvital tooth^d^	Burke and Lucarotti 2009	0.029	Recementation	0.20
			Repair	0.10
			Recrown^d^	0.60
			Extraction	0.10
Nonsurgical root canal treatment	Ng et al. 2008	0.085 (Ng et al. 2008)	Surgical retreatment	0.25
			Extraction	0.75
Surgical root canal treatment	Torabinejad et al. 2009	0.061	Extraction	1.00
Implant and implant-supported crown	Torabinejad et al. 2007	0.010	Recementation/refixing	0.60
			Recrown	0.20
			Reimplant	0.20

Analyses were performed for populations with low and high caries prevalence and risks, respectively. Sensitivities and specificities of caries detection with and without AI assistance were derived from our primary study and a meta-analysis as described. The probabilities of lesion development and progression if untreated or infiltrated were calculated according to patient’s age (α) using hazard functions. If possible, we calculated mean values and 95% confidence intervals or ranges to estimate distributions (in parentheses) for random sampling during microsimulation. Other transition probabilities were extracted from large cohort studies or systematic reviews; allocation probabilities were similarly derived from cohort studies or claims data analyses, or they were informed by clinical experience, as described in the main text and elsewhere.

AI, artificial intelligence; N/A, not applicable.

a In a sensitivity analysis, data from 7 independent dentists were used. Mean (minimum–maximum) sensitivities and specificities of these dentists were 0.36 (0.19–0.65) and 0.91 (0.69–0.98).

b Data from 15- to 19-y-olds. Risk of pulpal exposure during recomposite assumed to be 10%. Crowning assumed if rerestored before.

c Ninety-five percent of exposed pulps were treated using direct capping, and 5% were assumed to receive immediate root canal treatment.

d For nonvital crowned teeth, risk of endodontic complications was calculated separately (Ferrari et al. 2012).

### Health Outcomes, Costs, and Discounting

Effectiveness was measured as the mean time a tooth was retained (in years). We assumed the valuation attached to the various health states of retained teeth (e.g., nonrestored, filled, crowned tooth) to be identical in the absence of any further data in this direction. Cost calculations were based on the German public and private dental fee catalogues, Bewertungsmaßstab (BEMA) and Gebührenordnung für Zahnärzte (GOZ), as described in the Appendix (where both units and quantities are fully displayed for each course of treatment). The costs of applying AI for analyzing a pair of bitewings are currently not known and were set at 8 euro in the base case and varied in sensitivity analyses. All costs were estimated per simulated tooth; costs that occurred for more than 1 tooth (e.g., for radiographs, for AI, for assessment and advice) were distributed among the relevant teeth. Detailed estimates on costs, including an estimation of development and operational costs for such an AI application, can be found in the Appendix. Note that we did not reflect on the efficiency impact of using an AI application (i.e., the possible time savings).

Costs and effectiveness were discounted at 3% per annum (Institute for Quality and Efficiency in Health Care [IQWiG] 2009). Discounting accounts for time preference, with effectiveness gains or costs being valued higher if they are realized now than later. Discount rates were varied to explore the impact of higher or lower discounting. Given our study’s perspective, opportunity costs were not accounted for.

### Analytical Methods

To analyze the model, we performed Monte-Carlo microsimulations, with 1,000 independent teeth being followed over the mean expected lifetime of each individual in annual cycles. A microsimulation model was chosen, as this allows to follow individual teeth over time and has been the standard in similar studies in the field. Incremental cost-effectiveness ratios (ICERs) were used to express cost differences per gained or lost effectiveness when comparing the 2 strategies. To introduce parameter uncertainty, we randomly sampled transition probabilities from triangular or uniform distributions between calculated 95% confidence intervals (CIs) or the range of parameters (Briggs et al. 2002). Moreover, the probability that a strategy was acceptable to payers at different willingness-to-pay ceiling thresholds was explored (see the Appendix). Univariate sensitivity analyses were additionally performed.

## Results

### Study Parameters

The input parameters for our study are shown in Table 1. The sensitivity of visual-tactile detection was 0 for early lesions and remained low for advanced ones, while specificity was high. Using AI-based diagnostics assistance was consistently more sensitive than radiographic assessment by dentists without AI, while dentists showed a slightly superior specificity.

### Base-Case Scenario

**Figure 2.**
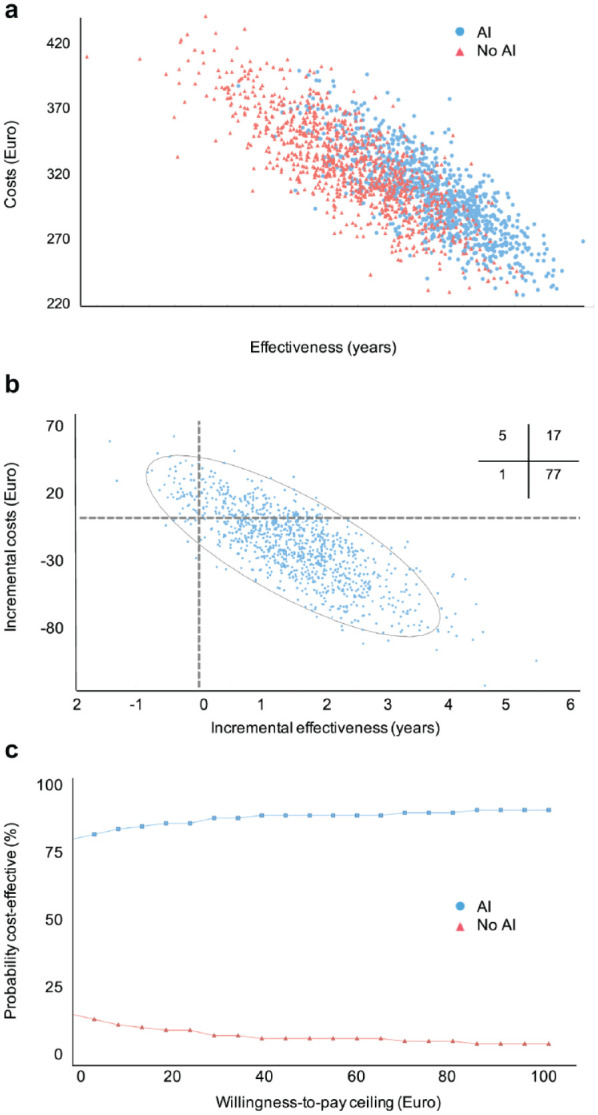
Cost-effectiveness plane, incremental cost-effectiveness, and net-benefit analysis of the base case. (**A**) The costs and effectiveness of the 2 comparators are plotted for 1,000 sampled individuals in each group. (**B**) The incremental costs and effectiveness of artificial intelligence (AI) compared with no AI are plotted. Quadrants indicate comparative cost-effectiveness (e.g., upper right: higher costs at higher effectiveness; lower right: lower costs and higher effectiveness). Inserted cross-tabulation: Percentage of samples lying in different quadrants. (**C**) We plotted the probability of comparators being acceptable in terms of their cost-effectiveness depending on the willingness-to-pay threshold of a payer. The range of willingness to pay was expanded from 0 to 100 euro and did not considerably change beyond this threshold.

In the base-case scenario (low risk, AI costs of 8 euro per application, dentists’ accuracy from meta-analysis), AI was more effective (tooth retention for a mean [2.5%–97.5%] 64 [61–65] y) and less costly (298 [244–367] euro) than conventional assessment by dentists (62 [59–64] y; 322 [257–394 euro]). The ICER was −13.9 euro/y (i.e., AI saved money at higher effectiveness). Figure 2 shows the cost-effectiveness plane (Fig. 2A), with AI being more effective and less costly in the majority of simulations. This was also reflected in the incremental cost-effectiveness plane (Fig. 2B), where most (>77%) cases found AI cost-effective (less costly, more effective). The cost-effectiveness increased even more for payers with a willingness-to-pay exceeding 0 euro/y (Fig. 2C).

### Sensitivity Analyses

A range of sensitivity analyses was performed (Table 2). In high-risk populations, the cost-effectiveness advantage of AI increased compared with the base case; this was also the case when using the reported dentists’ accuracy from the original diagnostic study in which AI and dentists were tested on the same imagery. If treating all detected lesions restoratively (also early ones), the cost-effectiveness was reversed; AI was more costly and less effective than no AI. If varying the costs for AI, the cost-effectiveness advantage of AI was only minimally affected. If not assuming to replace any lost teeth, the cost-effectiveness difference decreased, but AI remained the more cost-effective choice. Assuming all lost teeth to be replaced or varying the discount rates had only a limited impact on cost-effectiveness.

**Table 2.** Cost-effectiveness in the Base-Case and Sensitivity Analyses.

	Dentists with AI		Dentists without AI		
Analysis	Cost (Euro)	Effectiveness (y)	Cost (Euro)	Effectiveness (y)	ICER (Euro/y)
Base case	298 (244–367)	64 (61–65)	322 (257–394)	62 (59–64)	−13.9
High risk	402 (323–478)	61 (58–63)	482 (390–570)	58 (55–61)	−27.1
If treating only restoratively	468 (374–564)	56 (54–60)	321 (238–383)	62 (60–64)	−27.8
Dentists’ accuracy from Garcia-Cantu et al. (2020)	298 (244–367)	64 (61–65)	329 (236–402)	62 (59–64)	−15.5
Low costs for AI (4.00 euro/analysis)	296 (242–351)	64 (61–65)	322 (257–394)	62 (59–64)	−12.8
High costs for AI (12.00 euro/analysis)	301 (246–370)	64 (61–65)	322 (257–394)	62 (59–64)	−14.8
0% teeth replaced	246 (218–275)	64 (61–65)	249 (203–284)	62 (59–64)	−1.5
100% teeth replaced	310 (252–378)	64 (61–65)	339 (262–406)	62 (59–64)	−14.5
Discounting rate 1%	498 (394–627)	64 (61–65)	572 (407–701)	62 (60–64)	−35.9
Discounting rate 5%	209 (175–244)	64 (60–65)	214 (164–255)	62 (60–64)	−2.5

Mean and 2.5% to 97.5% percentiles are shown. The rationale behind modeling an upper/lower bound of AI costs of 4.00 and 8.00 euro is provided in more detail in the Appendix. The range of replaced teeth includes the minimum and maximum possible. The range of discounting rates follows recommendations for cost-effectiveness studies in our setting (IQWiG 2009).

AI, artificial intelligence; ICER, incremental cost-effectiveness ratio.

## Discussion

The number of studies using AI and, specifically, deep learning in dentistry and dental image analysis is rapidly increasing (Schwendicke, Golla, et al. 2019). So far, these studies largely have focused on evaluating the developed models for their accuracy, which, by itself, transports only limited value to patients, providers, and health care organizers. This value rather emanates from the subsequent decisions made, as these determine if, for example, a caries lesion is arrested or progressed and if a tooth is lost or retained long term, all of which come with associated costs (Baelum et al. 2012). The present study assessed the cost-effectiveness of an AI-based diagnostic assistance application for caries detection and found AI less costly and more effective over a lifetime horizon despite being more costly initially. We hence confirm our hypothesis.

A number of aspects need to be discussed. First, and as shown in other instances in dental cost-effectiveness analyses before, we found the initially more costly comparator to be cost-effective long term (Schwendicke et al. 2016). This was because costs occurring over the lifetime of a patient, mainly for restorative, prosthetic, or further care, are rather high and accumulate in an escalating fashion. The relevance of costs for tooth replacement, for example, was confirmed in a sensitivity analysis of the present study. Our findings highlight the relevance of an appropriate study horizon to yield meaningful results; a short-term follow-up study would not have been able to reflect this comprehensively. Second, this cost-effectiveness was realized largely via AI having a higher sensitivity, facilitating effective arresting therapies. Notably, the difference in cost-effectiveness between AI and no AI was not as pronounced as the difference in sensitivities (AI was by large twice as sensitive as the dentists) mainly due to reversed differences in specificity, in which the dentists showed a limited advantage. This demonstrates the effects false-positive diagnoses can have on cost-effectiveness and highlights the importance of the test specificity as a measure of accuracy. Our findings suggest the need of dentists doublechecking any AI suggestions. Third, and associated, the cost-effectiveness emanating from sensitivity and specificity stems from the deduced therapies; we have demonstrated in previous studies that combining a sensitive detection method is only cost-effective if “safe,” noninvasive, or microinvasive therapies are applied (Schwendicke, Paris, et al. 2015). Assigning invasive therapies to any (i.e., also early) detected lesion (as has been found the standard for many dentists worldwide) drastically changed the cost-effectiveness of AI: the assessment without AI showed nearly the same costs and effectiveness, mainly as the low sensitivity of dentists to detect early lesions allowed only very limited negative impacts of restoring these lesions. In contrast, AI was now by far more costly and less effective than assessment without AI. It seems recommendable that such sensitive AI applications provide information about not only the detected lesion but also its depth and recommended therapy options to mitigate the outlined detrimental effects of possibly harmful treatment decisions stemming from early lesion detection. Fourth, we found this cost-effectiveness to be modified by the risk profile of the population. In high-risk populations, it was more relevant to be sensitive (using AI) than in low-risk populations. Future AI applications should aim to reflect such risk profiles, for example, by cross-usage of meta-data of each practice (reflecting on prevalence) or risk profile data from each individual patient. Last, we found the cost-effectiveness ranking to hold true in a range of further sensitivity analyses. These reflected the uncertainties in costs but also accuracies, discount rates, and clinical pathways (via replacement probabilities for lost teeth). For costs, it was relevant to show that even for relatively high costs per AI application, cost-effectiveness was given. Notably, these costs are only assumptions right now and reflect the potential direct medical costs to providers; they are, however, grounded in credible assumptions toward the costs for developing and operating such an AI application, as detailed in the Appendix. Nonmedical or indirect costs (e.g., those for implementation, educating staff) were not considered. From a societal perspective, further costs (e.g., indirect costs to patients) may be relevant. Regarding the accuracies, we used different accuracy data for dentists and found the model to be cost-effective in both analyses. Notably, both analyses also showcased the discussed variability in dentists’ accuracy, which introduces uncertainty in cost-effectiveness (e.g., Fig. 2A). Future studies should aim to reflect these uncertainties in more depth and may be wanting to put monetary values on reducing them (e.g., through value-of-information analyses).

This study has a number of strengths and limitations. First, and as a strength, this is the first study assessing the cost-effectiveness of any “AI” or, specifically, deep learning intervention in dentistry. Our study thus provides, for the first time, a different perspective on the potential impact of this disruptive technology for dental care. It further can assist to inform decision makers in health care organizations but also research funding to prioritize (or not) further assessments of and investments into these technologies. Second, the used approach and the employed model have been validated before; they allow extrapolating short-term accuracy data into meaningful long-term outcomes (tooth retention and costs). Third, and as a limitation, the data informing our study were partially based on 1 specific diagnostic accuracy study, testing 1 deep learning model against a limited and likely nonrepresentative number of dentists. Comparing different models against different dentists may yield different outcomes. Fourth, our evaluation was focused on German health care and is not fully generalizable. This relates to both costs but also the modeled treatment decisions and further aspects such as assumptions toward tooth replacement or discounting. That said, cost estimation using fee items of the German fee item catalogues has been found to reflect the true treatment efforts to some degree and to yield estimates comparable with those from other health care settings (Schwendicke, Graetz, et al. 2014; Schwendicke et al. 2018). Moreover, they reflect the true direct medical costs to third party-payers and hence, indirectly, society and are relevant for decision makers in Germany. Also, while costs may somewhat differ in other health care systems, it is unlikely that the obtained ranking of interventions will change, but rather the magnitude of the observed differences. Last, and as mentioned, this study only partially reflects all aspects relevant to decision making; it is a simplification, and our findings should be confirmed by prospective randomized studies reflecting on the real-world decisions and cost-effectiveness emanating from the application of AI for diagnostic assistance in dentistry. For example, it is conceivable that dentists may not accept all detections and deviate in their treatment decisions, possibly to the benefit or detriment of patients’ health. Moreover, as outlined, treatment decisions will admittedly consider a range of factors (perceived lesion activity, patients’ wishes, dentists’ expertise), all of which will determine cost-effectiveness to some degree.

In conclusion, and within the limitations of this study, AI to support proximal caries detection on bitewings was cost-effective regardless of a payer’s willingness to pay. This cost-effectiveness was grounded in a higher sensitivity to detect early caries lesions, allowing to arrest them and thereby avoiding costly late retreatments. Notably, it only held true if detected early lesions were treated nonrestoratively. AI has the potential to improve care at lower health care costs.

## Author Contributions

F. Schwendicke, J. Krois, contributed to conception, design, data acquisition, analysis, and interpretation, drafted and critically revised the manuscript; J.G. Rossi, contributed to data acquisition, analysis, and interpretation, critically revised the manuscript; G. Göstemeyer, contributed to data acquisition and interpretation, critically revised the manuscript; K. Elhennawy, contributed to data analysis and interpretation, critically revised the manuscript; A.G. Cantu, S. Gehrung, contributed to data analysis, critically revised the manuscript; R. Gaudin, A. Chaurasia, contributed to data interpretation, critically revised the manuscript. All authors gave final approval and agree to be accountable for all aspects of the work.

## Supplemental Material

DS_10.1177_0022034520972335 – Supplemental material for Cost-effectiveness of Artificial Intelligence for Proximal Caries Detection Click here for additional data file. Supplemental material, DS_10.1177_0022034520972335 for Cost-effectiveness of Artificial Intelligence for Proximal Caries Detection by F. Schwendicke, J.G. Rossi, G. Göstemeyer, K. Elhennawy, A.G. Cantu, R. Gaudin, A. Chaurasia, S. Gehrung and J. Krois in Journal of Dental Research

## References

[bibr1-0022034520972335] Baelum V Hintze H Wenzel A Danielsen B Nyvad B. 2012. Implications of caries diagnostic strategies for clinical management decisions. Community Dent Oral Epidemiol. 40(3):257–266. 22103270 10.1111/j.1600-0528.2011.00655.x

[bibr2-0022034520972335] Briggs AH O’Brien BJ Blackhouse G. 2002. Thinking outside the box: recent advances in the analysis and presentation of uncertainty in cost-effectiveness studies. Annu Rev Public Health. 23(1):377–401. 11910068 10.1146/annurev.publhealth.23.100901.140534

[bibr3-0022034520972335] Burke FJT Lucarotti PSK. 2009. Ten-year outcome of crowns placed within the General Dental Services in England and Wales. J Dent. 37(1):12–24. 18487003 10.1016/j.jdent.2008.03.017

[bibr4-0022034520972335] Ferrari M Vichi A Fadda GM Cagidiaco MC Tay FR Breschi L Polimeni A Goracci C. 2012. A randomized controlled trial of endodontically treated and restored premolars. J Dent Res. 91(7 suppl):S72–S78. 10.1177/0022034512447949 22699672

[bibr5-0022034520972335] Frencken JE Innes NP Schwendicke F. 2016. Managing carious lesions: why do we need consensus on terminology and clinical recommendations on carious tissue removal? Adv Dent Res. 28(2):46–48. 27099356 10.1177/0022034516639272

[bibr6-0022034520972335] Garcia Cantu A Gehrung S Krois J Chaurasia A Gomez Rossi J Gaudin R Elhennawy K Schwendicke F. 2020. Detecting caries lesions of different radiographic extension on bitewings using deep learning. J Dent. 100:103425. 32634466 10.1016/j.jdent.2020.103425

[bibr7-0022034520972335] Gimenez T Piovesan C Braga MM Raggio DP Deery C Ricketts DN Ekstrand KR Mendes FM. 2015. Visual inspection for caries detection: a systematic review and meta-analysis. J Dent Res. 94(7):895–904. 25994176 10.1177/0022034515586763

[bibr8-0022034520972335] Husereau D Drummond M Petrou S Carswell C Moher D Greenberg D Augustovski F Briggs AH Mauskopf J Loder E. 2013. Consolidated Health Economic Evaluation Reporting Standards (CHEERS)–explanation and elaboration: a report of the ISPOR Health Economic Evaluation Publication Guidelines Good Reporting Practices Task Force. Value Health. 16(2):231–250. 23538175 10.1016/j.jval.2013.02.002

[bibr9-0022034520972335] Institute for Quality and Efficiency in Health Care (IQWiG). 2009. Appraisal of recommendations by the scientific board of IQWIG regarding “methods to assess cost-effectiveness in German public health insurance” [würdigung der empfehlung des wissenschaftlichen beirats des iqwig zur „methodik für die bewertung von verhältnissen zwischen nutzen und kosten im system der deutschen gesetzlichen krankenversicherung”] 2009 [accessed 2017 May 15]. https://www.iqwig.de/download/09-03-18_Wuerdigung_der_Empfehlung_des_Wissenschaftlichen_Beirats.pdf.

[bibr10-0022034520972335] Kassebaum NJ Smith AGC Bernabé E Fleming TD Reynolds AE Vos T Murray CJL Marcenes W Abyu GY Alsharif U, et al.; GBD 2015 Oral Health Collaborators. 2017. Global, regional, and national prevalence, incidence, and disability-adjusted life years for oral conditions for 195 countries, 1990–2015: a systematic analysis for the global burden of diseases, injuries, and risk factors. J Dent Res. 96(4):380–387. 28792274 10.1177/0022034517693566 PMC5912207

[bibr11-0022034520972335] LeCun Y Bengio Y Hinton G. 2015. Deep learning. Nature. 521(7553):436–444. 26017442 10.1038/nature14539

[bibr12-0022034520972335] Lumley PJ Lucarotti PSK Burke FJT. 2008. Ten-year outcome of root fillings in the General Dental Services in England and Wales. Int Endod J. 41(7):577–585. 18479376 10.1111/j.1365-2591.2008.01402.x

[bibr13-0022034520972335] Ng YL Mann V Gulabivala K. 2008. Outcome of secondary root canal treatment: a systematic review of the literature. Int Endod J. 41(12):1026–1046. 19133093 10.1111/j.1365-2591.2008.01484.x

[bibr14-0022034520972335] Pallesen U van Dijken JWV Halken J Hallonsten A-L Höigaard R. 2013. Longevity of posterior resin composite restorations in permanent teeth in public dental health service: a prospective 8 years follow up. J Dent. 41(4):297–306. 23228499 10.1016/j.jdent.2012.11.021

[bibr15-0022034520972335] Ronneberger O Fischer P Brox T. 2015. Dental X-ray image segmentation using a u-shaped deep convolutional network [accessed 2020 Oct 19]. http://www-o.ntust.edu.tw/~cweiwang/ISBI2015/challenge2/isbi2015_Ronneberger.pdf

[bibr16-0022034520972335] Schwendicke F Engel AS Graetz C. 2018. Long-term treatment costs of chronic periodontitis patients in Germany. J Clin Periodontol. 45(9):1069–1077. 29981185 10.1111/jcpe.12984

[bibr17-0022034520972335] Schwendicke F Golla T Dreher M Krois J. 2019. Convolutional neural networks for dental image diagnostics: a scoping review. J Dent. 91:103226. 31704386 10.1016/j.jdent.2019.103226

[bibr18-0022034520972335] Schwendicke F Graetz C Stolpe M Dorfer CE. 2014. Retaining or replacing molars with furcation involvement: a cost-effectiveness comparison of different strategies. J Clin Periodontol. 41(11):1090–1097. 25255893 10.1111/jcpe.12315

[bibr19-0022034520972335] Schwendicke F Meyer-Lueckel H Stolpe M Dörfer CE Paris S. 2014. Costs and effectiveness of treatment alternatives for proximal caries lesions. PLoS One. 9(1):e86992. 24475208 10.1371/journal.pone.0086992 PMC3903601

[bibr20-0022034520972335] Schwendicke F Paris S Stolpe M. 2015. Detection and treatment of proximal caries lesions: milieu-specific cost-effectiveness analysis. J Dent. 43(6):647–655. 25862278 10.1016/j.jdent.2015.03.009

[bibr21-0022034520972335] Schwendicke F Splieth C Breschi L Banerjee A Fontana M Paris S Burrow MF Crombie F Page LF Gaton-Hernandez P, et al. 2019. When to intervene in the caries process? An expert Delphi consensus statement. Clin Oral Investig. 23(10):3691–3703. 10.1007/s00784-019-03058-w 31444695

[bibr22-0022034520972335] Schwendicke F Stolpe M Innes N. 2016. Conventional treatment, hall technique or immediate pulpotomy for carious primary molars: a cost-effectiveness analysis. Int Endod J. 49(9):817–826. 26331379 10.1111/iej.12537

[bibr23-0022034520972335] Schwendicke F Stolpe M Meyer-Lückel H Paris S Dörfer C. 2013. Cost-effectiveness of one- and two-step incomplete and complete excavation. J Dent Res. 92(10):880–887. 23945975 10.1177/0022034513500792

[bibr24-0022034520972335] Schwendicke F Stolpe M Meyer-Lueckel H Paris S. 2015. Detecting and treating occlusal caries lesions: a cost-effectiveness analysis. J Dent Res. 94(2):272–280. 25503613 10.1177/0022034514561260 PMC4438735

[bibr25-0022034520972335] Schwendicke F Tzschoppe M Paris S. 2015. Radiographic caries detection: a systematic review and meta-analysis. J Dent. 43(8):924–933. 25724114 10.1016/j.jdent.2015.02.009

[bibr26-0022034520972335] Torabinejad M Anderson P Bader J Brown LJ Chen LH Goodacre CJ Kattadiyil MT Kutsenko D Lozada J Patel R, et al. 2007. Outcomes of root canal treatment and restoration, implant-supported single crowns, fixed partial dentures, and extraction without replacement: a systematic review.J Prosthet Dent. 98(4):285–311. 17936128 10.1016/S0022-3913(07)60102-4

[bibr27-0022034520972335] Torabinejad M Corr R Handysides R Shabahang S. 2009. Outcomes of nonsurgical retreatment and endodontic surgery: a systematic review. J Endod. 35(7):930–937. 19567310 10.1016/j.joen.2009.04.023

