# Supplementary material for: Cost-effectiveness of Artificial Intelligence for Proximal Caries Detection
Source: J Dent Res. 2020 Nov 16;100(4):369–76. doi: 10.1177/0022034520972335 (PMC7985854; doi:10.1177/0022034520972335)
Supplement: DS_10.1177_0022034520972335 – Supplemental material for Cost-effectiveness of Artificial Intelligence for Proximal Caries Detection [file DS_10.1177_0022034520972335.pdf]

Appendix

German healthcare

In Germany, the medical insurance is two-tiered, with most individuals (>87%) being publicly insured (statutory insurance) and only a minority being privately insured. For members of the statutory insurance, nearly all dental procedures are fully covered, while only few need to be partially or fully paid out-of-pocket or by private (additional) insurances.

Synopsis of the systematic review we have built our comparator on

Briefly, this review had included clinical or in vitro studies reporting on humans with primary caries lesions (clinical studies), or human teeth with primary natural caries lesions (in vitro studies), which were submitted to caries detection via radiographic means. Secondary lesions or teeth with artificially induced lesions were excluded. The caries status of the examined surface needed to be assessed using a reference test, i.e. a “gold standard”, which could have been destructive (histologic, microradiographic or operative assessment) or non-destructive (visual-tactile assessment without [occlusal] or with [proximal] tooth separation). Reported data should allow to construct a diagnostic 2×2 table. Electronic databases (Medline via PubMed, Embase via Ovid, Cochrane Central) had been screened for articles published until September 2014, without any further restriction regarding publication date or language. Diagnostic reviews were additionally searched via the Medion database.

Modelling period

The teeth were followed over the patients’ average lifetime, which was assumed to be 66 years from now on based on the life expectancy of males in Germany (deStatis 2017); note that this period of time only serves to determine the simulation period and would be minimal longer for females (without any relevant impact on outcomes).

Caries prevalence

In the base-case, a population with low caries prevalence and low risk of lesion development and progression was simulated; in a sensitivity analysis, a population with high prevalence and risk was assessed. The rationale for not modelling this uncertainty in one joint evaluation (e.g. using uncertainty distributions) was to display risk-specific cost-effectiveness, as this has been found relevant in the context of our study (Schwendicke et al. 2015a). Surface-prevalence of proximal caries lesions was estimated based on data from Sweden (Mejàre et al. 2004) as described before (Schwendicke et al. 2015a).

Cost estimation

German dentists use fee items to claim for reimbursement for dental treatments. For most dental procedures and patients, items will be drawn from the public catalogue BEMA. For few treatments (composite restorations in posterior teeth, implants and ISC), fees are usually derived from the private catalogue; publicly insured patients pay the additional costs ouf-of-pocket or via additional private insurers For GOZ, factoring of the chargeable item points is common to determine the fees of private treatment in Germany; we used the standard multiplication factor (2.3). Using fee items allowed us to estimate costs occurring to payers, which was in line with our study perspective (Schwendicke et al. 2013a).

BEMA defines fee items within the public insurance, which covers 87% of insured Germans (GKV-Spitzenverband 2013), with only few treatments not being fully covered or reimbursed. For these items, calculation was based on GOZ or “analogue-items”. BEMA points vary between federal states, insurers and treatment groups. For our cost calculation we used mean state points for the biggest insurer (AOK) from one federal state, Bavaria. For GOZ, point values were applied, with 0.0562421 Euro/point. Factoring of GOZ item-points was usually performed via the standard multiplication factor (×2.3). Certain positions (mainly radiographic assessments) are further coded in GOÄ (Gebührenordnung für Ärzte), with 9 GOÄ points equaling 1 BEMA point. Laboratory and material costs were estimated based on Laboratory Fee Catalogues (BEL II/BEB). Costs for BELII/BEB have been transformed into monetary values for the following tables. Items were restricted in number and character to reflect cost

limitations. Total costs per course of treatment were calculated based on the quantification (q) of itemized costs (c), i.e. $c_1 \times q_1 + c_2 \times q_2$ etc., and calculated in Euro. Details can be found further below.

Net benefit approach and cost-effectiveness acceptability

Using estimates for costs (c, in Euro) and effectiveness (e, in years), the net benefit of each strategy combination was calculated using the formula

$$\text{net benefit} = \lambda \times \Delta e - \Delta c,$$

with λ denoting the ceiling threshold of willingness to pay, i.e. the additional costs a decision maker is willing to bear for gaining an additional unit of effectiveness (Drummond et al. 2005). If $\lambda > \Delta c / \Delta e$, an alternative intervention is considered more cost-effective than the comparator despite possibly being more costly (Briggs et al. 2002). We used the net-benefit approach to calculate the probability of a detection strategy being acceptable regarding its cost-effectiveness for payers with different willingness-to-pay ceiling thresholds.

Detailed calculation of costs per course of treatment

Costs for visual-tactile detection were assumed to be based on BEMA 01. We assumed that the costs of a regular examination would be distributed over all examined teeth and expected an average of 28 teeth to be examined, i.e. BEMA 01 equaling 18.90 Euro divided by 28 = 0.68 Euro. Costs for radiographic assessment were estimated based on BEMA Ä925a. We assumed all posterior teeth to be assessed, i.e. costs were distributed over 16 teeth. Hence, GOÄ925a equaling 12.60 was divided by 16 = 0.79. The costs for the AI intervention were varied between 4.00 to 12.00 Euro per image analysis, as it is currently unclear which costs would be generated; the base-case costs were set at 8.00 Euro. AI costs were also distributed over the teeth as described. A more detailed assessment of AI costs can be found below. Infiltration was assumed to cost 84.99 Euro as described elsewhere (Schwendicke et al. 2014b). All other costs were estimated as follows.

(1) Costs for AI

We assumed the following costs to have occurred for establishing the AI intervention:

- Data generation and labeling: 3,686 images, 4 annotators, each annotation per image 5 min, average salary incl tax and social security contributions approx. 39,- Euro/h (TV-Ä Charité); in total 47.918 Euro
- Training of the AI model, approx. 4 months data science costs, at monthly costs for 5.750,- Euro (TV-E13), in total 23.000 Euro
- Software engineering costs for the intervention, approx. 12 months full time at 5.750,- Euro (TV-E13), in total 69.000 Euro
- Regulatory process incl. user tests, clinical evaluation, technical documentation and quality management system (might be available already, though), approx. 100,000 Euro
- Total development costs: 239,918 Euro. These were assumed to be distributed over the first 100,000 use cases, i.e. analyses, i.e. 2.40 Euro per image.

We further assumed costs per goods sold (COGS) to occur as follows:

- Cloud infrastructure. According to our simulations, costs of 1-3 Euro per image analysis occur depending on the load and the accepted waiting time for each dentist.
- Support etc. including possible step-by-step instructions; we assumed one monthly email and one monthly phone contact, each consuming 10 min of a support employee's time, i.e. 39,- Euro per hour (TV-E13), in total 13 Euro/ month. Assuming each dentist to use the intervention for each bitewing, at an estimated number of 20 bitewings per month, this summed up to 0.65 Euro per month.
- The total COGS hence summed up to 1.65-3.65 Euro/image.

Total costs per image were hence assumed to be 4.05 Euro to 6.05 Euro per analysis. We further assumed any provider of such a tool would also need to generate additional revenue for management, sales and marketing, development and profit (overhead), and assumed this block to account for 55% of the abovementioned costs (i.e. 2.24-3.33 Euro). Hence, total costs were assumed to range between 6.29-9.38 Euro. Given that developmental costs would, however, be diluted with each use case exceeding 100,000 analyses, and given that the costs for revenue, sales and marketing, development and profit would also be reduced if the intervention was scaled up in its use, our cost estimate between 4-12 Euro seemed to realistically cover possible cost scenarios.

(2) Direct capping and direct restoration

Treatment	Position BEMA/GOÄ/GOZ/L	Points	Number of Treatments	Euros
Clinical investigation	01	18	1	18.9
Sensitivity testing	8	6	1	6.3
Radiographic assessment	GOÄ925 a	12	1	12.6
Anesthesia	40/41a	8/12	1	10.5
Special measures during restorative therapy	12	10	1	10.5
Adhesive restoration, two surfaces	GOZ 2100	556	1	71.92
Liner	25	6	1	6.3
Direct capping	26	6	1	6.3
**Total**				**143.32**

(3) No capping, and direct restoration

Treatment	Position BEMA/GOÄ/GOZ/L	Points	Number of Treatments	Euros
Clinical investigation	01	18	1	18.9
Sensitivity testing	8	6	1	6.3
Radiographic assessment	GOÄ925 a	12	1	12.6
Anesthesia	40/41a	8/12	1	10.5
Special measures during restorative therapy	12	10	1	10.5
Adhesive restoration, two surfaces	GOZ 2100	556	1	71.92
Liner	25	6	1	6.3
**Total**				**137.02**

(4) Repair of existing restorations

Treatment	Position BEMA/GOÄ/GOZ/L	Points	Number of Treatments	Euros
Clinical investigation	01	18	1	18.9
Sensitivity testing	8	6	1	6.3
Radiographic assessment	GOÄ925 a	12	1	12.6
Anesthesia	40/41a	8/12	1	10.5
Filling, three surfaces	13c	39	1	42.90
**Total**				**91.20**

(5) Root-canal treatment (assumed three root canals)

Treatment	Position BEMA/GOÄ/GOZ/L	Points	Number of Treatments	Euros
Clinical investigation	01	18	1	18.9
Sensitivity testing	8	6	1	6.3
Radiographic assessment	GOÄ925 a	12	3	37.8
Anesthesia	40/41a	8/12	1	10.5
Rubber dam	12	10	3	31.5
Direct core build-up	13B	39	1	40.95
Vital pulp extirpation	28	18 per canal	3	56.7
Root canal treatment	32	29 per canal	3	91.35
Root canal filling	35	17 per canal	3	53.55
**Total**				**347.55**

(6) Full metal crown

Treatment	Position BEMA/GOÄ/GOZ/L	Points	Number of Treatments	Euros
Clinical investigation	01	18	1	18.9
Sensitivity testing	8	6	1	6.3
Radiographic assessment	GOÄ925 a	12	1	12.6
Anesthesia	40/41a	8/12	1	10.5
Special measures during restorative therapy	12	10	1	10.5
Temporary crown	19	19	1	16.75
Full metal crown	20a	148	1	130.53
Dental materials				22.07
Laboratory				
Situation model	0010	5.74	2	11.48
Used resin	0023	12.14	1	12.14
Single-tooth dye	0051	9.19	1	9.19
Occludator	0120	8.42	1	8.42
Full-metal crown	1021	72.27	1	72.27
Non-precious metal alloy	9700	11.68	1	11.68
Delivery	9330	3.98	3	11.94
**Total**				**365.27**

(7) Post-core crown

Treatment	Position BEMA/GOÄ/GOZ/L	Points	Number of Treatments	Euros
Clinical investigation	01	18	1	18.9
Sensitivity testing	8	6	1	6.3
Radiographic assessment	GOÄ925 a	12	1	12.6
Post–core metal	18b	80	1	84
Temporary post	21	28	1	24.69

Temporary crown	19	19	1	16.75
Recementation of temporary crown	24c	7	1	7.35
Full metal crown	20a	148	1	130.53
Dental materials				22.07
Laboratory				
Situation model	0010	5.74	2	11.48
Used resin	0023	12.14	1	12.14
Single-tooth dye	0051	9.19	1	9.19
Occludator	0120	8.42	1	8.42
Metal post casting	1050	44.92	1	44.92
Full-metal crown	1021	72.27	1	72.27
Non-precious metal alloy	9700	11.68	1	11.68
Delivery	9330	3.98	3	11.94
**Total**				**505.23**

(8) Recementation

Treatment	Position BEMA/GOÄ/GOZ/L	Points	Number of Treatments	Euros
Clinical investigation	01	18	1	18.9
Sensitivity testing	8	6	1	6.3
Radiographic assessment	GOÄ925 a	12	1	12.6
Recementation of a crown	24a	25	1	26.25
**Total**				**64.05**

(9) Non-surgical root canal retreatment

Treatment	Position BEMA/GOÄ/GOZ/L	Points	Number of Treatments	Euros
Clinical investigation	01	18	1	18.9
Sensitivity testing	8	6	1	6.3
Radiographic assessment	GOÄ 5000	50	3	19.40
Anesthesia	GOÄ 0090/0100	60/70	1	8.41
Rubber dam	GOZ 2040	65	3	25.23
Root canal treatment	GOZ 2410	392 per canal	3	152.13
Irrigation	GOZ 2420	70	3	27.15
Microscopy	GOZ 0110	400	3	155.20
Root canal filling	GOZ 2440	258 per canal	3	100.11
Medication	GOZ 2430	204 per visit	3	79.17
**Total**				**592**

(10) Surgical root canal retreatment

Treatment	Position BEMA/GOÄ/GOZ/L	Points	Number of Treatments	Euros
Clinical investigation	01	18	1	18.9
Sensitivity testing	8	6	1	6.3
Anesthesia	40/41a	8/12	1	10.5
Radiographic assessment	GOÄ925 a	12	2	25.2
Apicectomy	54b	96	1	100.8
Retrograde filling	35	17 per canal	1	17.85
**Total**				**179.55**

(11) Tooth/implant removal

Treatment	Position BEMA/GOÄ/GOZ/L	Points	Number of Treatments	Euros
Clinical investigation	01	18	1	18.9
Sensitivity testing	8	6	1	6.3
Radiographic assessment	GOÄ925 a	12	2	25.2
Anesthesia	40/41a	8/12	1	10.5
Extraction of multirooted tooth	44	15	1	15.75
**Total**				**76.65**

(12) Implant insertion

Treatment	Position BEMA/GOÄ/GOZ/L	Number of Treatments	Euros
Initial charting and consultation	GOÄ1	1	10.72
Intra-oral investigation	GOÄ6	1	13.41
Detailed consultation	GOÄ3	1	20.11
Cost estimation	GOZ 0030	1	25.87
Panoramic radiograph	GOÄ 5004	2	107.24
Diagnostic models	GOZ 0050	1	15.52
Radiographic diagnosis and guide	GOZ 9000	1	114.35
Use of radiographic guide	GOZ9003	1	12.94
Implant insertion	GOZ 9010	1	199.86
Implant		1	131.86
Suture material		1	7.68
Post-operative care	GOZ 3300	2	16.82
Prescription and medication	GOÄ 70	1	5.36
Topical anesthesia	GOZ 0080	2	7.76
Anesthesia	GOÄ 0090/0100	2	16.82
Implant re-exposure	GOZ 9040	1	80.98
Gingiva-former		1	25.70
Laboratory			
Situation model	0002	3	28.08
Replica	0241	1	15.20
Occludator	0402	1	10.16
Diagnostic wax-up	0832	1	10.30

Positioning splint	1224	1	60.20
Radiographic guide	1311	1	3.92
Delivery	0701	3	17.82
**Total**			**958.68**

(13) Implant-supported porcelain-bonded crown

Treatment	Position BEMA/GOÄ/GOZ/L	Number of Treatments	Euros
Crown preparation	GOZ 2200	1	171.01
Temporary crown	GOZ 2270	1	34.93
Manipulation of abutments	GOZ 9050	2	80.98
Individual impression	GOZ 5170	1	32.34
Dental materials			22.07
Laboratory			
Situation model	0010	3	17.22
Individual tray	0211	1	19.51
Used resin	0023	1	12.14
Single-tooth dye	0051	1	9.19
Occludator	0120	1	8.42
Gingival mask	0223	1	10.99
Working with a supra-structure	2971	1	25.77
Working on abutment	2973	1	47.47
Crown core	2122	1	70.26
Porcelain coverage	2612	1	99.83
Non-precious metal alloy	9700	1	11.68
Delivery	9330	6	25.98
Impression post	9237	1	56.54
Laboratory implant	9238	1	25.40
Abutment and screw	9239	1	84.82

**Total**			**866.55**
